# Sensory integration of a light touch reference in human standing balance

**DOI:** 10.1371/journal.pone.0197316

**Published:** 2018-06-06

**Authors:** Lorenz Assländer, Craig P. Smith, Raymond F. Reynolds

**Affiliations:** 1 Sensorimotor Performance Lab, University of Konstanz, Konstanz, Germany; 2 School of Sport, Exercise & Rehabilitation Sciences, University of Birmingham, Birmingham, United Kingdom; Ludwig-Maximilians-Universitat Munchen, GERMANY

## Abstract

In upright stance, light touch of a space-stationary touch reference reduces spontaneous sway. Moving the reference evokes sway responses which exhibit non-linear behavior that has been attributed to sensory reweighting. Reweighting refers to a change in the relative contribution of sensory cues signaling body sway in space and light touch cues signaling finger position with respect to the body. Here we test the hypothesis that the sensory fusion process involves a transformation of light touch signals into the same reference frame as other sensory inputs encoding body sway in space, or vice versa. Eight subjects lightly gripped a robotic manipulandum which moved in a circular arc around the ankle joint. A pseudo-randomized motion sequence with broad spectral characteristics was applied at three amplitudes. The stimulus was presented at two different heights and therefore different radial distances, which were matched in terms of angular motion. However, the higher stimulus evoked a significantly larger sway response, indicating that the response was not matched to stimulus angular motion. Instead, the body sway response was strongly related to the horizontal translation of the manipulandum. The results suggest that light touch is integrated as the horizontal distance between body COM and the finger. The data were well explained by a model with one feedback loop minimizing changes in horizontal COM-finger distance. The model further includes a second feedback loop estimating the horizontal finger motion and correcting the first loop when the touch reference is moving. The second loop includes the predicted transformation of sensory signals into the same reference frame and a non-linear threshold element that reproduces the non-linear sway responses, thus providing a mechanism that can explain reweighting.

## Introduction

### Influence of light finger touch on balance

Spontaneous sway in human upright stance is reduced when touching a space-stationary reference with a finger [1,2]. The amplitude of touch force in ‘light touch’ experiments is defined as a contact which is insufficient to stabilize the body mechanically, and is usually specified as less than 1 N. Thus, the sway-reducing effect can be attributed to integration of the touch reference as an additional source of sensory information. The emphasis on a sensory effect is supported by the finding that the sway reduction disappears when the forearm and hand are anesthetized by ischemic compression of the upper arm [3]. In addition to haptic cues (contact forces), a sensation of the finger position with respect to the body is required [2,4]. For a space-stationary touch reference, any change in finger position relative to the body is directly related to postural sway, and this can be exploited to stabilize the body.

### Reweighting of light touch cues

When moving the touch reference, light touch cues signal a superposition of body and touch reference motion. Since humans use light touch cues to control balance, moving the touch reference evokes body sway [5,6]. This suggests that the stimulus motion is falsely interpreted by the central nervous system (CNS) as self-motion. This can also explain why sway becomes entrained with a partner during interpersonal light touch [7]. The sway responses increase in magnitude with stimulus amplitude. However, this increase is non-linear; the ratio between evoked sway and stimulus amplitude becomes smaller with increasing stimulus amplitude [8,9]. This non-linear behavior has been attributed to a change in the contribution of light touch cues, referred to as sensory reweighting [10]. To achieve reweighting, the neural control mechanism needs to integrate the light touch reference with other sensory cues. However, the neural mechanisms underlying the integration process remain largely unknown and raise some fundamental questions how light touch cues are combined with other sensory variables.

### Geometric considerations

To identify whether a change in finger position relative to the body is due to self or touch reference motion, the nervous system needs to compare light-touch cues to other cues signaling body sway. When closing the eyes, the main additional sensory contributions are the proprioceptive reference to the floor and the vestibular reference to gravito-inertial space [11]. In upright stance, both sensory systems signal body sway around the ankle joints, i.e. angular signals which are used to stabilize the body center of mass [12]. In order to fuse sensory cues with each other and quantify sensory conflicts, these cues should be in the same reference frame. Thus, light touch cues need to be transformed to a variable that is comparable to the angular cues signaling body sway in space, or vice versa. This could be achieved by the brain taking the geometric configuration of the body into account. The hypothesis of the present study is the existence of a mechanism that performs such a transformation.

The experimental setup to test the hypothesis evolved from a consideration of the change in the relative positions of the finger and body that results from angular body sway around the ankle joints. During anterior-posterior sway, the finger moves along an arc, where the absolute motion largely depends on the radius given by the distance between the ankle joints (the center of rotation) and the finger. To test our hypothesis, we assumed that light touch is integrated as a variable encoding angular movement around the ankle joints. Assuming this assumption holds, the sway responses should depend only upon angular stimulus motion, and should be independent of the radial distance of the touch reference from the ankle joint. Thus, the prediction is that a moving tactile stimulus will evoke the same amplitude of sway when it is presented at two different heights, when the stimulus amplitudes are matched for angular motion around the ankle joints.

To maintain balance during light touch, the CNS must distinguish between stimulus and self-motion. Otherwise, large stimulus amplitudes could endanger postural stability. Therefore, some mechanism is required to down-weight the contribution of light touch cues in such situations. Such a mechanism has been discussed in earlier studies describing non-linearities in sway responses to light touch stimuli [8–10], but the precise nature of this mechanism has not been identified. The above-formulated geometric considerations predict that a transformation between body-finger cues and cues signaling angular sway around the ankle joints is involved in the reweighting mechanism.

### Identification of control models for human standing balance

To test the above predictions, system identification methods can be applied to test different model structures. In this approach, small stimuli that mechanically perturb balance or evoke a conflict between sensory signals are used to probe the dynamics of the human balance control mechanism [13,14]. In a seminal study, Peterka [12] used pseudo-random stimuli with broad frequency spectra to measure the characteristics of sway responses to tilts of the support surface and the visual scene. These sway response characteristics provided a sufficient basis for the identification of a control model. The model identified by Peterka [12] provided insight into basic properties of the human balance control mechanism. Key conclusions of this approach are 1) the sufficiency of sensory feedback despite long time delays, when using a PD controller with a loop gain about 1.3 times larger than required to counteract gravity, 2) an apparent time delay of 0.1–0.2 s, which depended on the stimulus amplitude, 3) an additional feedback component reducing body lean at very low frequencies (see also [15]), and 4) a change in sensory contributions depending on stimulus amplitudes, referred to as sensory reweighting.

To investigate the sensory integration of light touch cues, a similar approach can be used. Since touch reference motion evokes sway responses, it can be used to probe the dynamics of the control mechanism. Using a similar stimulus to that proposed by Peterka [12], at several stimulus amplitudes, should thus provide a broad characterization and may allow us to identify a quantitative model for the sensory integration of light touch cues. The quantitative model can then be used to test the above-formulated hypothesis on the sensory integration of light touch cues.

### Experiments and results

To test our hypotheses, we used a combination of experiments and model simulations. The experiments used touch reference motion stimuli of different amplitudes, applied at two different heights while the evoked body sway was measured. In contrast to our first prediction, the results suggest that humans primarily stabilize body-finger distance during light touch, without transforming the touch signal to an angular variable. Furthermore, our results suggest a mechanism that transforms other sensory cues (e.g. proprioception & vestibular information) from angular ankle-based coordinates into expected body-finger translation. This is in full agreement with the hypothesized transformation of sensory cues to comparable physical variables. The model simulations suggest that the expected body-finger distance is then compared to the actual body-finger distance encoded by light touch cues to generate reweighting. As such, the identified mechanism is reminiscent of the ‘Disturbance Estimation and Compensation’ concept [16–18].

## Materials and methods

### Subjects

Eight subjects (4 male, 4 female) with average height 175 +/- 11 cm, mass 70.5 +/- 13.7 kg and aged 27 +/- 2.5 years participated in the study. The study was approved by the University of Birmingham Ethics Committee, with subjects giving their written informed consent prior to the experiments.

### Experimental setup

Subjects stood barefoot with feet together on a force plate (Kistler 9286AA, Kistler Instrumente AG, Switzerland). With the right thumb and index finger, they lightly gripped a force sensor (F306 Disc Loadcell, Novatech Measurements Ltd., UK) attached to the end-point of a robotic device (HapticMaster, Moog FCS, Netherlands). The force sensor allowed for the measurement of grip force. The robotic device was movable in 3 dimensions, and was placed such that the zero position of the touch reference was 35 cm in front of the ankle joints and 35 cm to the right from the middle of both feet. Two electromagnetic motion sensors (Fastrak, Polhemus Inc., USA) were placed at hip and shoulder level to record body sway. Stimulus, actual touch reference motion, touch force, body sway, and ground reaction forces were recorded at 50 Hz using a PC with an AD converter (NI PCI-6220, National Instruments Corporation Ltd., UK).

#### Stimuli

**Fig 1:**
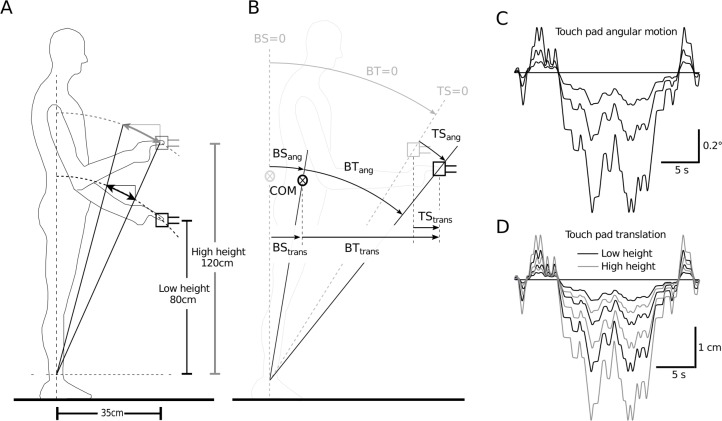
Experimental setup. A) Schema of the experimental setup with the two touch reference heights (not scaled proportionally). Arrows indicate touch reference motion. B) Definition of variables body-in-space (BS; body sway) and touch reference-in-space (TS; the stimulus), and body-to-touch reference (BT; light touch sensory input) as angle (_*ang*_) and horizontal translation (_*trans*_). C) Angular motion of the stimulus, where the stimulus is pairwise identical across touch reference heights. Shown are single cycles at three amplitudes; cycles were repeated 12 consecutive times within one experimental trial. D) Stimulus shown as horizontal motion, where the stimulus within one angular amplitude changes with touch reference height.

The robotic device was programmed to move in a small circular arc in the sagittal plane, with the center of the circle at the ankle joints (ankle height of 10 cm was assumed for all subjects to simplify the setup, Fig 1A). Three motion sequences with different angular amplitudes were each tested at a low height of 80 cm (conditions L1, L2, L3) and a high height of 120 cm above the ankle joints (conditions H1, H2, H3), resulting in a total of six conditions. One stimulus cycle comprised a 24-s long pseudo-random ternary sequence with 80 steps of zero, positive, or negative velocity [12,19]. Velocities were scaled to generate the predefined angular peak-to peak amplitudes (L1 = H1 = 0.29°, L2 = H2 = 0.57°, and L3 = H3 = 1.15°; Fig 1C). The sequence was repeated 12 consecutive times for each condition (amplitude x height), resulting in six trials with a length of 288s. While stimulus amplitudes were pairwise identical for the two heights in terms of angular motion, their absolute horizontal and vertical motion differed considerably (Fig 1D).

#### Calibration routine and calculation of center of mass sway

The primary output of the experiment was whole body center of mass (COM) movement without feet. The COM was derived from the motion of the hip and trunk sensors, by initially calibrating their motion against the center of pressure (COP) as recorded by the force plate. During very slow movement, the COP can be used as a projection of the COM [20]. Subjects performed a 2-min long calibration routine, moving very slowly forward and backward, bending both the ankle and hip joints [12]. Using a linear regression between hip and ankle marker and the center of pressure trajectories, calibration factors were obtained from which the anterior-posterior position of the COM during dynamic experimental trials was calculated based on the marker data. Angular COM sway was then obtained using anthropometric data and the relative COM height [21].

#### Procedures

Markers were attached to a corselet (shoulder marker) and at the approximate height of the greater trochanter (hip marker). After performing the calibration routine as described above, six experimental trials were run in random order, with a brief break in between trials. Before each trial subjects were instructed to close their eyes, stand comfortably upright and hold the robotic device lightly between their index finger and thumb without evoking large forces. Measured grip force was below 1N with Mean ± SD across trials 0.54 ± 0.38 N. To distract subjects from the balance task, and to reduce auditory orientation cues, subjects listened to non-musical audio (talk radio) via headphones.

### Data analysis

All analysis and model simulations were performed using Matlab and Simulink (The Mathworks, Natick, USA). Two different stimulus-response combinations were used during the analysis: 1) angular stimulus (*TS*_*ang*_) and angular COM motion (*BS*_*ang*_); 2) horizontal translation of the stimulus (*TS*_*trans*_), along with horizontal translation of the COM (*BS*_*trans*)_. The first cycle of each trial was discarded to avoid transient effects. For body sway, the mean was subtracted from each cycle to avoid an influence of drifts on the data. Across the remaining 11 cycles of each subject, the arithmetic mean was calculated for each point in time. Since the stimulus is identical for each cycle repetition, the arithmetic mean provides an estimate of the body sway response to the stimulus reducing the effect of random sway components. To test the angular integration hypothesis, the root-mean square (RMS) of the angular sway response was calculated for each subject and compared between the two heights. For equal angular stimuli, identical RMS values would indicate similar sway response amplitudes and therefore an independence between the two heights. The analysis was repeated comparing body sway (COM) and stimulus as horizontal translation, instead of angular movement.

A two-way repeated measures ANOVA implemented in JASP (JASP Team 2016, Version 0.7.5.6) was used to statistically test effects of height and angular stimulus amplitude on angular body sway. A one-way repeated measures ANOVA was used to test the effect of horizontal stimulus amplitude on horizontal body sway responses, where a statistical test of height was not possible. The reason is that horizontal displacement multiplied by height provides pairwise identical samples and therefore a pairwise linear dependence between the stimuli at the low and high height.

For a more detailed characterization of the sway responses and for model-based interpretations, the spectral characteristics of the translational sway responses were analyzed. To this end, the spectra of individual translational body sway and recorded stimulus cycles were calculated using the Fast-Fourier-Transform implemented in the function ‘fft’ in Matlab. The arithmetic mean of the spectra was calculated across cycles and subjects. Averaging across subjects introduces inter-individual variability in the data. However, it also reduces overall variability, which was required given the low signal power at higher frequencies. Frequency response functions were calculated for each experimental condition as the ratio between averaged translational sway response spectra and averaged translational stimulus spectra [14]. These complex valued functions were displayed in terms of bode diagrams and provide a frequency and stimulus-dependent non-parametric characterization of the human balance control mechanism, including biomechanics. For the bode diagrams and model simulations, only odd harmonics in the frequency range of 0.04 to 0.8 Hz were considered, since pseudo-random ternary sequences have no stimulus energy at even harmonics, and declining signal power at higher frequencies. Finally, magnitude-squared coherence was calculated. Cross power spectra and power spectra of stimulus and sway response as obtained from the ‘fft’ function were calculated and averaged across subjects and cycles. Coherence was calculated as the squared, averaged cross power spectrum, divided by the product of averaged stimulus and response power spectra and displayed across frequency [14].

### Model simulations

The experimental frequency response functions provide insight into the dynamics of human sway responses to touch-motion stimuli across a wide range of frequencies, and several stimulus amplitudes. Thus, they provide a solid basis to identify a parametric control model using simulations and parameter optimization procedures. Input and output of the model and for the calculation of experimental frequency response functions were the horizontal translation of the touch reference (*TS*_*trans*_) and horizontal COM displacement of body sway (*BS*_*trans*_, compare Fig 1B). The model structure is described in two steps. Building blocks from the literature are described below, while the feedback mechanism for the integration of light touch cues as identified in the current study are described in the Results section.

#### Plant / Physics

The human body was modeled as a single inverted pendulum with inertia *J* and gravitational forces proportional to angular orientation of the body times body mass *m*, gravitational constant *g* and body COM height *h* and without taking Coriolis forces into account. While this simplification does not capture the dynamics of the human body fully, it is proven to be a useful approximation for identifying the main characteristics of sensory integration [12]. Furthermore, small angle approximations (sin α = tan α = α) were used to calculate the gravitational torque and to calculate horizontal body-finger distance from body lean in space.

#### Sensors

Our model assumes that horizontal body-finger distance, as well as the body angle relative to the space vertical are available to the brain through sensory cues. Sensors in the model refer to complex sensory systems with a neural integration of multiple transducer signals and across multiple joints. The underlying assumption is a reconstruction of the body kinematics that have evoked the transducer signals. While neural correlates of kinematic variables have been found in the spinal cord and cerebellum of cats and rats [22,23], using physical variables rather than transducer signals also is a conceptual step that simplifies model simulations [12,17,24,25]. Sensors *BT*_*trans*_ (Body-to-Touch reference) refer to the proprioceptive cues that encode the horizontal distance between touch reference and body COM and its velocity (also see Discussion for a more detailed consideration). Both are assumed to have ideal transfer characteristics within the dynamic range of the experiment (indicated by a gain of one). Sensors *BS*_*ang*_ (Body-in-Space) refer to the vestibular-derived angular body orientation in space AND the proprioceptive orientation with respect to the floor. Since the floor was space-stationary in our experimental setup, both these sensory systems signal the same physical quantity and are lumped together as one signal in the model. Sensors *BS*_*ang*_ also encode position and velocity cues and are assumed to have ideal dynamics.

#### Balance control without light touch

The sensory cues of Sensors *BS*_*ang*_ are fed back and amplified by a neural PD controller to generate the torque driving the body [12,18]. The feedback mechanism contains a time delay to account for neural conduction, neural processing, and muscle activation delays. One additional contribution to the overall torque is a low-pass filtered torque feedback that was proposed to account for human compensation of prolonged body lean, i.e. a sway reduction at low frequencies [15]. The output of the PD controller provides the torque that is driving the body and in interaction with the Physics/Plant results in changes of body orientation again sensed by the Sensors.

#### Sensory integration of light touch cues

The structure and parameters of the neural control model for the integration of light-touch cues were subject to the analysis in the current study. The structure was inspired by the Disturbance Estimation and Compensation (DEC) concept [16,17,26] and derived heuristically (see Results and Discussion). The model was formulated and simulations were run in Simulink (The Mathworks, Natick, USA). Model parameters were obtained using parameter optimization procedures, where parameters of the model formulated in Simulink were adjusted to minimize a predefined objective function. The objective function is given by

$$err=\sum_{f,c}\frac{{\left|{FRF}_{exp}(f,c)-{FRF}_{}(f,c)\right|}^{2}}{f}$$

where FRF_exp_ and FRF_sim_ are the complex valued frequency response functions from the experiments and the model simulations, respectively. The objective function contained a sum across all frequencies *f* and conditions *c*, such that one set of parameters was obtained for all six experimental conditions. The objective function was minimized using the ‘trust-region-reflective’ algorithm as implemented in the function ‘lsqnonlin’ of the Matlab Optimization Toolbox (The Mathworks, Natick, USA). The function was run several times manually changing starting parameters to avoid local minima. Several parameters were fixed during the optimization procedure. These parameters were the biomechanical parameters (*J*, *m*, *g*, *h*), which were derived from subjects’ anthropometrics and using anthropometric tables [21], the height of the touch reference (*hTP*), which was given by the experimental setup, and the low-pass filter constant *Flp* of the torque feedback. The latter was fixed to a value obtained from the literature [27], since the stimulus sequence does not provide sufficient information at low frequencies, to identify this parameter. All other parameters, namely the proportional and derivative overall loop gains *Kp* and *Kd*, the lumped time delay *dt*, the force feedback loop gain *Glp*, the light touch loop gain *LT*, and the estimator gain and threshold *Ge*, and *Te*, were subject to the optimization procedure.

Variance accounted for (VAF) was calculated for each experimental condition to assess the quality of the model quantitatively. VAF was calculated from the squared difference between experimental sway responses across cycles and subjects *e(t)* and the simulation output *s(t)* in the time domain. For both sequences *e(t)* and *s(t)*, the mean was subtracted before calculating VAF using

$$VAF=\left(1-\frac{\sum_{t}{\left|e(t)-s(t)\right|}^{2}}{\sum_{t}{\left|e(t)\right|}^{2}}\right)*100$$

### Random sway

As an additional source of information, also random sway, defined as the sway component not correlated with the stimulus, was analyzed. An estimate of random sway in the time domain can be assessed by calculating the standard deviation across cycles for each point in time. The RMS of the standard deviation was calculated for each subject and the same statistical analysis was performed as for the body sway responses.

## Results

### Angular stimuli and sway responses

**Fig 2:**
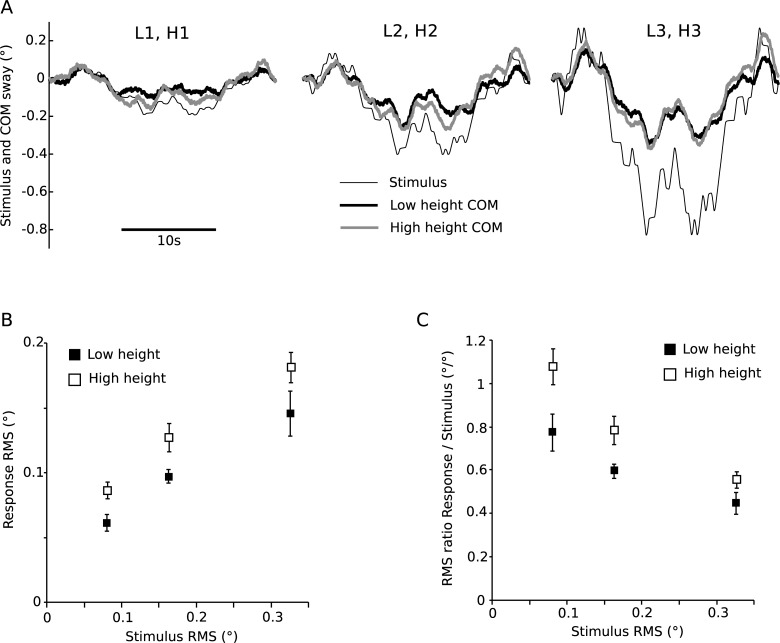
Angular body sway responses. A) Angular body sway responses (*BS*_*ang*_) to the stimulus applied at two heights (L, H) and three amplitudes (1, 2, 3), averaged across stimulus cycles and subjects, shown in the time domain. B) Root mean square (RMS) values and standard errors (SE) across subjects of the sway responses plotted against stimulus RMS. C) RMS and SE of the ratio between sway response RMS and stimulus RMS against stimulus RMS.

Fig 2 shows mean angular stimulus and COM angular response at the low and high touch reference heights for three stimulus amplitudes. The time series (Fig 2A) show that the COM followed the general pattern of the stimulus. The magnitude of COM angular response (Fig 2B) increased with stimulus amplitude for both the low and high conditions (F(2,14) = 37.50, p < 0.001). Although stimulus angle was the same for both touch reference heights within each stimulus amplitude, the magnitude of the angular COM response was significantly greater when the touch reference was at the high height (F(1,7) = 11.69, p = 0.01).

The ratio of COM response RMS and stimulus RMS (Fig 2C) was reduced with increasing stimulus amplitude for both the low and high touch reference, with a significant main effect of stimulus amplitude (F(2,14) = 29.29, p < 0.001). The RMS ratio was significantly greater in the high touch reference position compared to the low position for equal stimulus amplitudes (F(1,7) = 13.25, p = 0.01). Furthermore, the RMS ratio declined with increasing stimulus amplitude, which is indicative for a non-linearity in the underlying control mechanism.

### Translational stimuli and sway responses

**Fig 3:**
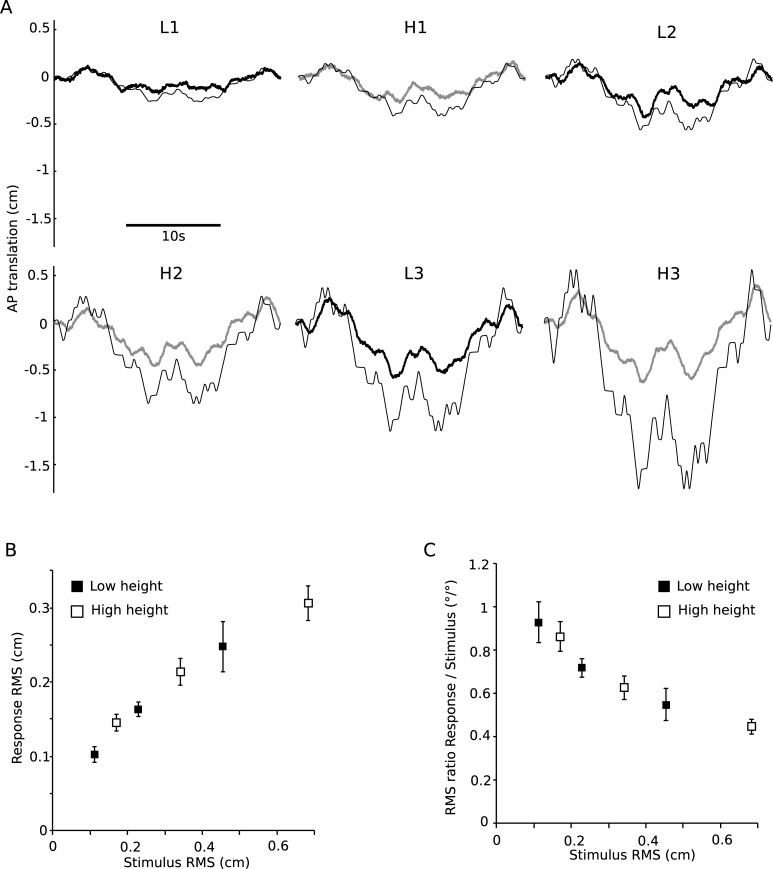
Horizontal translation of body sway responses. A) Horizontal translation of body sway responses (*BS*_*trans*_) to the stimulus applied at two heights (L, H) and three amplitudes (1, 2, 3), averaged across stimulus cycles and subjects, shown in the time domain. B) Root mean square (RMS) values and standard errors (SE) of sway responses plotted against stimulus RMS. C) RMS and SE of the ratio between sway response RMS and stimulus RMS against stimulus RMS.

After discovering an effect of touch reference height on angular COM responses for identical angular stimulus amplitudes (Fig 2), a second analysis step investigated whether this was an effect of greater absolute horizontal stimulus movement at the high height compared to the low height. In terms of absolute horizontal stimulus translation, six stimulus amplitudes were effectively tested (Fig 1D). Fig 3A shows mean across subjects of stimulus translation and COM translation for the six stimulus amplitudes. The magnitude of COM translation increased with greater stimulus translation (Fig 3B), with a significant effect of stimulus amplitude on COM translation amplitude (F(5,35) = 20.84, p < 0.001). The RMS ratio reduced with increased stimulus translation amplitude (Fig 3C), with a significant main effect of stimulus amplitude on COM translation response (F(5,35) = 11.53, p < 0.001).

The analysis of the translational components was conducted in the post-hoc analysis after rejecting the main hypothesis. The stimulus design did not allow a statistical analysis of the effect of height on the translation data. The reason is that translation stimulus amplitudes are pairwise linearly related through the touch reference height. However, translational response RMS values showed a gradual increase with translational stimulus amplitudes. No systematic difference, where sway responses of the low and high height are systematically shifted to each other were visible, indicating that sway responses were independent from the height of the touch reference.

### Model structure

**Fig 4:**
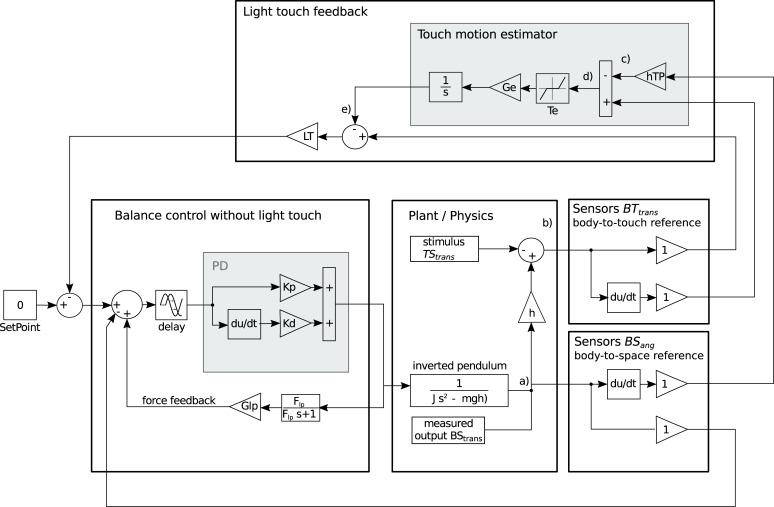
Sensory integration model for light touch cues. Triangles are gain factors, where values are given in Table 1 with the exception of the COM height h (0.96 m) and the touch reference height hTP (0.8 m or 1.2 m). Linearized inverted pendulum dynamics are implemented as a transfer function using the Laplace transform. ‘Set point’ provides the desired body orientation, which is adjusted by the light touch feedback. a-e indicate points of interest in the model. a) output of the inverted pendulum dynamics is the body angle in space. b) the sum calculates the physical horizontal distance between the finger and the body COM as it is sensed by light touch. c) the gain factor transforms the body angle as sensed by Sensors *BS*_*ang*_ to a horizontal distance ${\hat{BT}}_{trans}$ signal using the small angle approximation. d) the sum provides a sensory reconstruction of the Stimulus. e) indicates the correction of the light touch feedback through the estimated touch motion. The model is drawn to represent the signal flow in the central nervous system as compared to an engineering view with stimulus as positive input on the left and body sway as output for systems identification on the right.

The model that was identified to best describe the data shown in Fig 4. ‘*Plant’*, ‘*Sensors’*, and ‘*Balance control without light touch’* were formulated based on earlier studies (see Methods). The identified structure for the ‘*Light touch integration’* (Fig 4) consists of two components. The first component is a direct feedback of the horizontal body-finger distance amplified by a factor LT. The direct feedback of the horizontal component reproduces the experimentally found independence of the sway responses from the touch reference height shown in Fig 3. The second component is the ‘*Touch motion estimator’*, which was inspired by the tilt estimator of the DEC concept [16,17]. The estimator fuses velocity cues of body-finger distance from Sensors ${\dot{BT}}_{trans}$ and velocity cues of angular body sway obtained from Sensors *BS*_*ang*_. To allow a meaningful fusion, the angular Sensor *BS*_*ang*_ velocity signals are transformed to a translational variable ${\hat{\dot{BS}}}_{trans}$ (gain factor *hTP* and using the small angle approximation). After the transformation, the signal is identical to the change in body-finger distance, when the touch-pad is stationary. In other words, the transformed signal represents the change in body-to-touch reference distance as expected from body sway velocity ${\hat{\dot{BT}}}_{trans}$ (Fig 4C) sensed by Sensors *BS*_*ang*_. This expected ${\hat{\dot{BT}}}_{trans}$ signal can now be subtracted from the actual ${\dot{BT}}_{trans}$ signal from light touch (sensed by Sensors *BT*_*trans*_). The difference between these two signals provides a reconstruction of the horizontal finger velocity and therefore the light touch stimulus velocity $\hat{\dot{TS}}$ (Fig 4D).

The estimate is based on noisy sensory cues and not ideal with 1) limited sensitivity and 2) an undershooting where the estimated motion is only partly compensated. In agreement with the DEC concept [16], these imperfections in the human balance control mechanism can be mimicked in model simulations by 1) a threshold and 2) a gain factor smaller than one, respectively. The estimated touch-motion is then used to compensate the erroneous feedback from light touch. The estimator is effective through a subtraction of the estimated touch pad motion from the light-touch feedback whenever the signal exceeds the threshold; it partly corrects the light touch feedback. The threshold introduces a non-linear behavior that reproduces the experimentally observed amplitude non-linearity (see simulation results).

### Frequency domain analysis of experimental data

**Fig 5:**
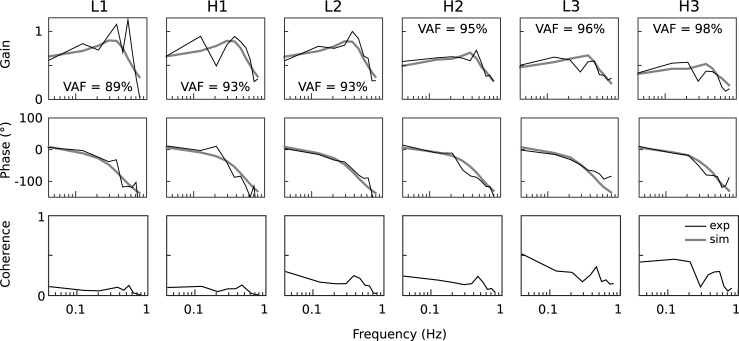
Frequency domain results. Frequency response functions of horizontal COM and stimulus motion displayed as gain (top row) and phase (mid row) against frequency for all six experimental conditions. Gain here is the amplitude ration between body sway response and stimulus. Phase is the temporal relation. The bottom row shows the magnitude-squared coherence against frequency. Shown are group averages of experimental results (black) and simulation results (grey).

**Fig 6:**
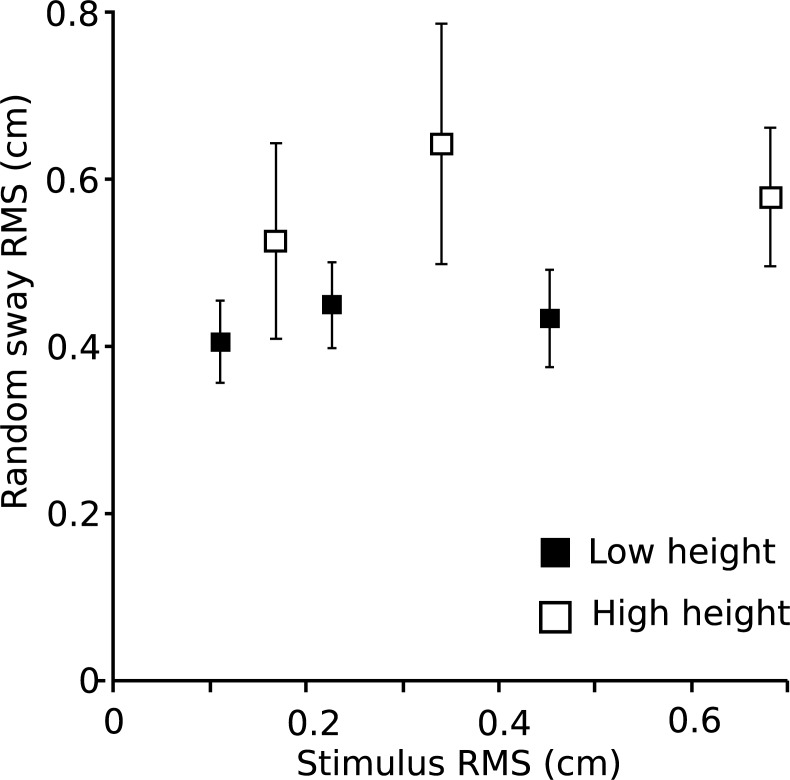
Random sway. Random sway RMS +/- SE against stimulus amplitude; a measure of the sway component not correlated with the stimulus.

Fig 5 shows the experimental frequency response functions in terms of gain and phase together with the results of the model simulations. Due to the finding that humans use translation cues rather than an angular interpretation of light touch cues, horizontal COM and stimulus displacement were used to calculate the frequency response functions. Gain is the amplitude ratio between horizontal body sway responses and the stimulus motion across frequency. Gain curves show a plateau in the frequency range of 0.1–0.5 Hz and a decline at higher and lower frequencies for all stimulus conditions. The differences across stimulus conditions, with a non-linear increase of sway responses with increasing stimulus amplitudes, were already described in Fig 3, however, without resolving sway responses across frequencies. Gain curves showed some fluctuations across frequencies that can be attributed to the small signal power of the stimulus, especially at higher frequencies, and were not considered physiologically relevant. The effects of these fluctuations on the model fits are further reduced, since parameters were fitted across all frequencies and stimulus conditions. Phase reflects the temporal relation between sway responses and stimuli across frequency. Phase values showed a phase-lead of about 10° at 0.04 Hz and a decrease with increasing frequency, reaching a phase lag of -100° between 0.5–0.8 Hz. There was no apparent difference in phase across amplitudes or touch-reference heights. Coherence can be interpreted as the signal-to-noise ratio [14], which corresponds to the relation between random sway and sway responses to the stimulus. Coherence values were generally very low, which was expected due to the small stimulus amplitudes that were applied. Coherence also showed a decline across frequency in all conditions and dropped to zero in the frequency range of 0.6–0.8 Hz. The small coherence values were also in agreement with the difference between the RMS values of sway responses and random sway (compare Figs 3B and 6).

### Simulation results

An optimization approach was used to identify the set of parameters that best reproduced the experimental frequency response functions. Fig 5 shows the simulation results for the best fit obtained from the optimization procedure. Gain and phase curves of the simulations resemble the main characteristics of the experimental frequency response functions. Notably, also the change in gain across stimulus amplitudes, and therefore the main non-linear characteristic, was reproduced well by the simulations. Coherence values were not calculated since random sway was not taken into account in the model simulations. Finally, Fig 5 also shows the variance accounted for (VAF) as a qualitative assessment of the model performance. The VAF ranged from 89% in the low to 98% in the high amplitude conditions and was 94% on average across conditions.

Table 1 shows the identified parameters together with parameters that were fixed (see Methods). Parameters *Kp*, *Kd*, and *Glp* were similar to parameters reported in the literature, despite using a different perturbation (i.e. light touch, instead of surface tilts; compare [12,27]). The time delay *dt* was also in the range reported in earlier studies (0.1–0.2 s in [12]). Parameters of the light touch feedback and the touch-reference motion estimator are also shown in Table 1. Light-touch feedback gain (LT*Kp) was 25% of the feedback gain without light touch (*Kp*). The estimator parameters were also in a feasible range, with the gain factor Ge smaller than one and the velocity threshold *Te* in the range of the stimulus velocities.

**Table 1:** Model parameters.

**Fixed Parameters**	COM height h* (m)	Body mass m* (kg)	Inertia J* (kg m^2^/s^2^)	Force feedback filter constant *Flp*
	0.96	68.5	76.4	20
**Balance control**	Proportional loop gain *Kp* (mgh)	Derivative loop gain *Kd* (mgh)	Force feedback loop gain *Glp* (N^-1^m^-1^)	Lumped time delay *dt* (s)
Light touch experiments	1.19	0.30	0.0021	0.106
Peterka tilt experiments	1.3**	0.47**	0.0017***	0.1–0.2**
**Light touch feedback**	Loop gain *LT*	Estimator gain *Ge*	Estimator threshold *Te* (°/s)	Simulation error
	0.29	0.58	0.0029	7.77

* Without feet, COM height above ankle joints

** adapted from [12]

*** adapted from [27]

### Random sway

The random sway component of horizontal body sway is plotted against stimulus magnitude in Fig 6 (RMS ±SE). There was a tendency for random sway to be larger in the high (compared to low) touch reference condition for all three stimulus amplitudes. This trend contrasts with the horizontal sway responses to the stimuli, where no effect of height was found. Statistical tests of the effects were not possible due to the same pairwise linear dependence of horizontal stimuli at the two heights.

## Discussion

### Comparison of angular and translational results

Three angular stimulus amplitudes were applied at two heights of the touch reference, and the evoked sway responses were compared. Identical angular stimulus amplitudes evoked sway responses which were significantly larger in the high height conditions as compared to the low height conditions (Fig 2). This finding rejects the hypothesis of an integration of light touch cues in the form of angular sway around the ankle joints. In contrast, plotting the RMS of the horizontal translation (Fig 3) showed a consistent increase of sway responses with stimulus amplitude, independent from stimulus height. This finding suggests that light touch cues are integrated as the horizontal distance of the finger with respect to the body COM.

The experimental results and the model-based interpretation provide consistent evidence for the idea that light touch cues are integrated as horizontal body-finger distance. However, other solutions might also be possible if they encode a geometric variable that is similar to the horizontal. For example, the absolute hip-finger distance or the horizontal shoulder-finger distance would only slightly differ from the horizontal body COM-finger distance. Such differences cannot be separated based on the presented experimental setup, which was designed to test whether humans integrate light touch cues as an angular variable.

Different touch heights might have also led to geometric differences in the arm configuration. Although arm kinematics were not measured, a theoretical analysis of the geometric changes showed that subjects had larger changes in shoulder angle at the high position, as compared to the low position. Since our results suggest that the touch height has no effect on sway responses, the integration of light touch cues seems to be independent from the joint angular configuration.

In summary, the presented approach is not able to determine which exact reference is encoded by light touch cues. However, our result strongly suggest that it is a horizontal kinematic variable and that it is independent from the geometric configuration of the arm.

### Non-linear increase of translational body sway responses

Sway response amplitudes increased with the horizontal translational stimulus amplitudes (Fig 3B). In agreement with earlier studies [8,10], the increase was non-linear, where changes in response amplitudes became smaller with increasing stimulus amplitudes. This behavior was confirmed by the RMS ratio (Fig 3C), which would remain at a constant level across stimulus amplitudes in a linear system but showed a gradual decline. The decrease in the RMS ratio points to a reweighting mechanism, where the contribution of light touch cues is reduced with increasing stimulus amplitudes. To know when to down-weigh the contribution of light touch cues, the brain needs to determine whether changes in body-finger position are due to ego-motion, or motion of the stimulus. Therefore, the non-linear sway responses also suggest that light touch cues are fused with other sensory cues which provide additional information on body sway. The nature of this fusion mechanism has been analyzed using model simulations.

### Model-based interpretations

The experimental results provide a broad characterization of human sway responses across frequency, six stimulus amplitudes, and two heights (Fig 5). The model proposed in this study (Fig 4) was able to reproduce the averaged sway responses of all experimental conditions with a variance accounted for of 94%, demonstrating a broad descriptive power (Fig 5). The integration of light touch in the model structure contains two functional components. First, a direct feedback mechanism where light touch cues are used as an input to the balance control mechanism. Thereby, the neural controller maintains the body at a fixed distance from the finger (Fig 4 ‘*Light touch feedback’*). Second, a mechanism that automatically reduces the influence of the light touch feedback, when the touch reference is sensed as moving in space (Fig 4 ‘*Touch motion estimator*’).

#### Light touch feedback

The light touch feedback is a simple negative feedback loop, which amplifies the sensory light touch cues with the loop gain *LT*. The consistent pattern of sway responses when considering the stimulus as horizontal motion (Fig 3) led us to also use the horizontal body-finger distance for this feedback loop. The gain factor *LT* determines the loop gain and therefore the control dynamics for the integration of light touch cues. A high loop gain on the one hand yields a more accurate tracking of the stimulus, since a change in sensory input leads to a larger corrective torque. On the other hand, a very high loop gain requires more effort, can lead to overshooting in dynamic situations and may even endanger control stability.

The body-finger distance is here modeled as the horizontal distance between whole body COM and the finger. This choice, however, is arbitrary in the sense that any horizontal kinematic variable could be encoded by the light touch cues. The reason is that the horizontal displacement between the finger and any given reference point within the body scales with the height of the reference point. Since the horizontal displacement (which depends linearly on the height of the internal reference point) is multiplied by the estimated gain factor *LT*, choosing a different reference height would merely lead to a different gain factor *LT*, maintaining the overall feedback loop gain.

#### Touch motion estimator

The negative feedback alone would lead to a linear increase in sway response amplitude with increasing stimulus amplitude. Since a linear increase is not in agreement with human behavior, our results support the existence of a reweighting mechanism that reduces the effect of light touch cues with increasing stimulus amplitude. Earlier studies suggest that humans use a reconstruction of external disturbances in the control of upright stance [16–18,26]. Following this idea, the model (Fig 4) contains a reconstruction of the stimulus. The reconstruction is based on the horizontal body-touch reference cues from light touch (*BT*_*trans*_) and cues signaling body-sway in space (*BS*_*ang*_). To fuse these signals, the brain needs to transform the cues signaling body sway—here the proprioceptive reference to the floor and the vestibular gravito-inertial reference—into an expected horizontal body-finger distance. Using the transformation allows for subtraction of the actual change from the expected change in body-finger distance, where the difference is a reconstruction of the horizontal finger movement in space (sum in the *Touch motion estimator*, Fig 4). In other words, the transformation to an expected body-finger distance is necessary to determine whether a change in body-finger distance is due to body sway or due to a moving reference. In the model, expected horizontal body-finger velocity ${\hat{\dot{BT}}}_{trans}$ is obtained by the multiplication of the velocity signal ${\dot{BS}}_{ang}$ with the touch height (gain factor *hTP*, which substitutes ‘*hTP* *sin(*BS*_*ang*_)’ using the small angle approximation).

The conversion from angular lean to horizontal distance is a step that needs to be actively implemented by the brain and has some interesting consequences. First, it requires that the brain knows the actual height of the touch reference and is able to use it to amplify a sensory signal. Second, extracting the height and using a horizontal kinematic variable in the control mechanism also requires the knowledge of vertical and horizontal body orientation. The role of the subjective vertical and horizontal in human perception and postural control is well founded [28]. The presented results add to this evidence, where the proposed combination of experiments and model simulations may provide new possibilities to further investigate the role of the subjective vertical in a feedback control mechanism. Third, in terms of control dynamics, changes in height and the resulting change in the gain factor *hTP* means a change in the loop gain of the velocity signal from Sensors *BT*_*trans*_. This aspect might be related to the results of the random sway analysis as discussed below.

### Random sway

In addition to analyzing the evoked sway response, we also analyzed the random sway component unrelated to the stimulus. All neural processes, sensory cues and muscle contractions contain noise and inaccuracies [29]. It was found that internal noise can explain spontaneous sway characteristics of standing humans [30,31], indicating an important role of noise in human balance control. Noise, together with the inherent instability of upright stance and the dynamics of the neural balance control mechanism thus provides a consistent explanation for random sway in upright stance.

Our results showed a significant effect of height but not of amplitude for random sway, where random sway was larger in the high conditions (Fig 6). The finding is in contrast to the findings of Rogers et al. [32], who reported a larger reduction in sway amplitudes for a touch reference at the shoulder as compared to one at knee level. However, Rogers et al. [32] investigated quiet stance, while random sway as presented in Fig 6 was obtained from perturbed stance.

To model the effect of different noise sources quantitatively in a non-linear model largely increases the complexity and is not covered in this study. However, the model and the results allow some qualitative considerations. Given the observed reduction of spontaneous sway [1,2], light touch may develop its effect in quiet stance through a reduction of noise. In terms of the model, this means that the corresponding sensory cues (*BT*_*trans*_) contain less noise as compared to the other sensory systems (*BS*_*ang*_). As described above, a change in height of the touch reference changes the loop gain of the velocity signal of Sensors *BS*_*ang*_ (gain factor *hTP* in the *Touch motion estimator*). This change in loop gain also amplifies noise present in this signal, and would lead to an increase in random sway with stimulus height, as observed in our results. However, it should be noted that the noise only becomes effective when stimulus and superimposed noise cross the threshold. This would also predict that the noise shows a dependency on the stimulus amplitude, if the stimulus is so small that stimulus and noise only partly cross the threshold. Such an effect can neither be confirmed nor rejected by our results, but requires further experiments. In the absence of a stimulus, i.e. with a stationary touch pad the threshold blocks most of the noise and random sway would further decrease.

In summary, changing the height of the touch reference leads to a change in loop gain when calculating the expected touch reference motion. The change in loop gain would then amplify the noise stemming from the proprioceptive reference to the floor and vestibular cues, thus leading to a change in random sway in dependence on touch reference height. Notably, this change in random sway depends on the activity of the *Touch motion estimator* (i.e. super threshold signals) and is not expected in spontaneous sway.

### Limitations

#### Horizontal stimulus

The study tested whether humans integrate light touch stimuli as angular body rotation around the ankle joints using rotational stimuli. The results clearly rejected the hypothesis and a second analysis step was added to further investigate our findings. The interpretation that humans use the horizontal movement of the stimulus when integrating light touch cues is therefore explorative and needs to be verified in future studies. Also the model-based interpretation of our results rests on the assumption that light touch is integrated as horizontal body-finger distance. However, the ability of the model to accurately reproduce the experimental data supports the hypothetical interpretation that horizontal body-finger distance is the variable encoded by light touch.

#### Model

The model was inspired by earlier quantitative models of perturbed upright stance and provides a consistent explanation of our experimental findings. An underlying assumption of our model (and others) is that the central nervous system integrates different sensory inputs into a single reference frame for controlling balance. While this seems like the most parsimonious control principle, we cannot rule out multiple sensorimotor control processes operating in parallel. An obvious limitation of the model is that it does not contain noise, leaving the observed random sway unexplained. Importantly, noise, stimulus characteristics and the overall dynamics of the system interact with the non-linear threshold that explains the changes in sway response amplitudes dedicated to reweighting. Future approaches should aim to include noise in the simulations to provide an explanation of sway responses to the stimulus and random sway. Finally, the model structure as presented in Fig 4 is not a unique solution in that small structural changes (e.g. changing gain factor *h* in *Plant/Physics* to *hTP*) provide only slightly worse simulation results. Thus, more experimental data is required to further refine the model.

#### Tracking mechanism

Light touch requires an additional task for the arm and possibly also the upper body to maintain the low touch forces at the contact point. The reason is that with just a passive connection to the touch reference the connection would maintain a stiffness from the stabilization of the arm. When body sway changes the distance between finger and touch reference, the stiffness would generate a force and the force limit of 1 N is quickly exceeded. As a consequence, the instruction to subjects to maintain a low force at the contact point results in an active tracking of the touch reference. This tracking is partly visible in the stabilization of the body with respect to the touch reference. However, additional corrections of the arm likely exist due to the much smaller inertia of the arm as compared to the large inertia of the body.

Some studies suggest that the forces exerted at the touch reference are major contributors in the integration of the light touch cues [4]. From our perspective, the tracking mechanism needs to exploit these force cues (whether vertical or shear forces) to maintain the precondition of a light touch. However, the results from the model simulations suggest that the kinematics of body orientation with respect to the finger are used for the stabilization of the whole body COM. In summary, the tracking mechanism is not taken into account by the model and might provide additional insights into the mechanism underlying the integration of light touch cues.

#### Movement perception

Previous research on visually-evoked postural responses suggests a connection between conscious motion perception and the strength of the evoked sway response [33]. In our experiment, the extent to which conscious awareness of object motion relates to the postural response is uncertain. Subjects were consistently aware of touch reference motion for the largest motion stimulus, but they were less certain for the smallest stimulus. It may be that conscious perception directly mirrors the evoked postural response i.e. when the sensorimotor system misinterprets object-motion as self-motion, thus evoking a strong postural response, this would be accompanied by a lack of conscious awareness of object motion. Conversely, as touch pad motion increases in amplitude and frequency, and the gain of the postural response declines, this may correspond to increased awareness. However, a better understanding of the precise interplay between perception and balance control would require a more detailed psychophysical investigation.

### Summary

The study shows that sway responses to a moving reference are independent from touch height, while random sway not related to the stimulus appears to become larger with increasing height. Additional analysis steps suggest that light touch cues are integrated as horizontal body COM movement with respect to the finger (i.e. the touch reference). A quantitative model for the integration of light touch cues was presented. The model simulations suggest that the central nervous system converts sensory cues to comparable variables prior to the fusion process. The model further provides a potential explanation for reweighting of light touch cues through a non-linear threshold mechanism in the sensory reconstruction of the stimulus.

## Supporting information

S1 Dataset Sensory integration of a light touch reference. Each file provides the time traces of the whole body center of mass or the stimulus for one individual experimental trial of one subject. Each row contains one of the eleven cycle repetitions recorded within each trial and used for analysis.
(ZIP)
